# Donepezil enhances understanding of degraded speech in Alzheimer's disease

**DOI:** 10.1002/acn3.471

**Published:** 2017-09-27

**Authors:** Chris J. D. Hardy, Yun T. Hwang, Rebecca L. Bond, Charles R. Marshall, Basil H. Ridha, Sebastian J. Crutch, Martin N. Rossor, Jason D. Warren

**Affiliations:** ^1^ Dementia Research Centre Department of Neurodegenerative Disease Institute of Neurology University College London London United Kingdom

## Abstract

Auditory dysfunction under complex, dynamic listening conditions is a clinical hallmark of Alzheimer's disease (AD) but challenging to measure and manage. Here, we assessed understanding of sinewave speech (a paradigm of degraded speech perception) and general cognitive abilities in 17 AD patients, before and following a 10 mg dose of donepezil. Relative to healthy older individuals, patients had impaired sinewave speech comprehension that was selectively ameliorated by donepezil. Our findings demonstrate impaired perception of degraded speech in AD but retained perceptual learning capacity that can be harnessed by acetylcholinesterase inhibition, with implications for designing communication interventions and acoustic environments in dementia.

## Introduction

Alzheimer's disease (AD) is associated with impaired higher cortical auditory function and communication, particularly in complex, dynamic acoustic environments, and the role of hearing impairment in cognitive decline is currently the focus of intense interest.^1^ Patients with AD frequently struggle to understand spoken information in the presence of background noise or when delivered in unfamiliar accents or voices,^2^, ^3^, ^4^ restricting communication and quality of life. Auditory dysfunction has been identified as a harbinger of incipient dementia in AD.^5^ The role of acetylcholine in auditory function is highly pertinent, particularly for dynamic perception under difficult or changing listening conditions or where there is a requirement for auditory plasticity.^6^ Cholinergic system degeneration is core to the disease process in AD and the most widely used symptomatic therapies (the acetylcholinesterase inhibitors) promote the effects of endogenous acetylcholine.^7^, ^8^ Cholinergic modulation impacts early auditory processing in healthy older people.^9^ However, currently, there is little information about the effects of cholinergic modulation on auditory function and communication in AD. Modulation of cholinergic transmission in AD has been shown to boost both verbal memory and auditory cortical function.^10^ Speech perception is therefore an attractive target for acetylcholinesterase inhibition in AD; indeed, procholinergic verbal memory benefit may depend in part on enhanced sensory encoding of speech.

Here, we assessed degraded auditory perception and the effect of increasing acetylcholinesterase inhibition in patients with AD versus untreated healthy older people, using the classical paradigm of sinewave speech.^11^ Sinewave transformation reduces speech signals to a series of ‘whistles’ (corresponding to formant contours) from which spectral detail has been stripped. Normal naïve listeners rapidly and spontaneously learn to understand sinewave messages.^12^ We hypothesized that compared with untreated healthy controls, AD patients in a relative cholinergic deficiency state (prior to the next dose of acetylcholinesterase inhibitor) would show impaired understanding of sinewave speech but that this would improve disproportionately to other cognitive functions following a dose of acetylcholinesterase inhibitor, given that sinewave speech perception is likely to depend on cognitive plasticity and dynamic neural processing.

## Methods

### Participants

Seventeen patients with typical amnestic AD (eight female; mean age 71.8 years) and 17 healthy older individuals (six female; mean age 66.6 years) participated. All patients fulfilled consensus criteria for AD^13^ of mild to moderate severity and all were established on Donepezil 10 mg/day when studied. No participant had a clinical history of hearing impairment and participants abstained from caffeinated beverages during the study. Demographic and clinical data for all participants are summarized in Table 1. All participants gave informed consent to participate in the study. Ethical approval was granted by the London‐Bromley Research Ethics Committee, in accordance with Declaration of Helsinki guidelines.

**Table 1:** General and behavioral test performance characteristics in the participant groups

Characteristic	Session	Healthy controls	AD	Group comparison	
				Statistic	*P*‐value
General					
Gender (M:F)	NA	11:6	9:8	*χ* ^2^ = 0.49	0.486
Age (years)	NA	66.6 (7.3)	71.8 (8.2)	t = 1.93	0.062
Handedness (R:L)	NA	16:1	17:0	NA	NA
Education (years)	NA	16.5 (1.8)	15.6 (2.6)	t = −1.13	0.267
MMSE (/30)	NA	29.9 (0.2)	23.9 (3.3)	*z* = −4.99	<0.001
Symptom duration (years)	NA	NA	4.5 (2.7)	NA	NA
Donepezil duration (months)	NA	NA	20.8 (13.1)	NA	NA
Antidepressant therapy (no.)^a^	NA	2	2	NA	NA
Behavioral tests					
*General cognitive*					
RMT Faces (/25):	baseline	24.4 (0.9)	19.7 (3.6)^b^	*z* = −3.61	<0.001
	change	0.3 (1.0)	0.0 (3.3)	*z* = 0.89	0.375
RMT Words (/25)	baseline	24.4 (1.5)	18.0 (3.4)^b^	*z* = −4.55	<0.001
	change	−0.1 (0.8)	0.4 (3.1)	*z* = 0.77	0.443
GNT (/30)	baseline	25.5 (3.6)	14.1 (8.3)	*z* = −4.18	<0.001
	change	0.8 (1.5)	−0.6 (2.5)	*z* = −1.81	0.071
WASI matrices (/32)	baseline	25.5 (4.0)	11.6 (6.8)	*z* = −4.42	<0.001
	change	1.2 (2.7)	−0.6 (3.4)	*z* = −1.40	0.163
BPVS (/51)	baseline	49.1 (1.7)	40.4 (10.1)^b^	*z* = −4.00	<0.001
	change	−0.5 (1.2)	0.6 (2.3)	*z* = 2.09	0.038
*Sinewave speech experiment*					
Sinewave speech (/20)	baseline	15.4 (2.5)	10.3 (5.1)	*z* = −3.40	<0.001
	repeat	16.3 (2.7)	13.9 (3.7)	*z* = −1.82	0.069
	change	0.9 (3.1)	3.6 (3.7)^c^	*t* = −2.36	0.025
Session change score^d^	baseline	2.0 (1.5)	2.7 (2.8)	*t* = −0.91	0.369
	repeat	2.4 (2.1)	2.5 (1.4)	*t* = −0.19	0.849
	change	0.4 (2.2)	−0.2 (3.3)	*t* = 0.61	0.544
Clear speech control (/10)	baseline	10.0 (0.0)	9.9 (0.2)	NA	NA
	repeat	10.0 (0.0)	10.0 (0.0)	NA	NA

**Figure 1:**
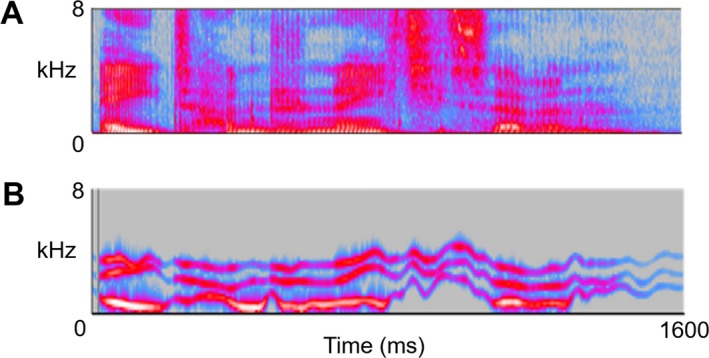
The spectrograms show examples of the stimuli used to assess sinewave speech processing. **A:** natural (i.e., clear) three‐digit spoken number (‘eight hundred and eighty‐seven’). **B:** the corresponding sinewave replica. Frequency is depicted on the *y*‐axis, in kilohertz (kHz) and time is depicted on the *x*‐axis, in milliseconds (msec); the sinewave replica retains the centre frequencies of the formant contours but omits the spectral detail evident in natural speech. Numbers were spoken in quiet by a young adult male speaker in a standard Southern English accent, and recorded as digital wavefiles (sampling rate 44.1 kHz, mean (SD) duration 1485 (111) msec) using Audacity^®^ software; sinewave stimuli were generated from the natural speech recordings using a procedure under Praat^®^ software (http://www.lifesci.sussex.ac.uk/home/Chris_Darwin/Praatscripts/SWS) and root‐mean‐square intensity was fixed for all stimuli. During testing, all stimuli were administered in a quiet room via ATH‐M50X Audio‐Technica^®^ headphones, at a comfortable listening level (at least 70dB) for each participant. Participants were instructed that on each trial they would hear a distorted three‐digit number and asked to write down the number as fully as possible.

**Figure 2:**
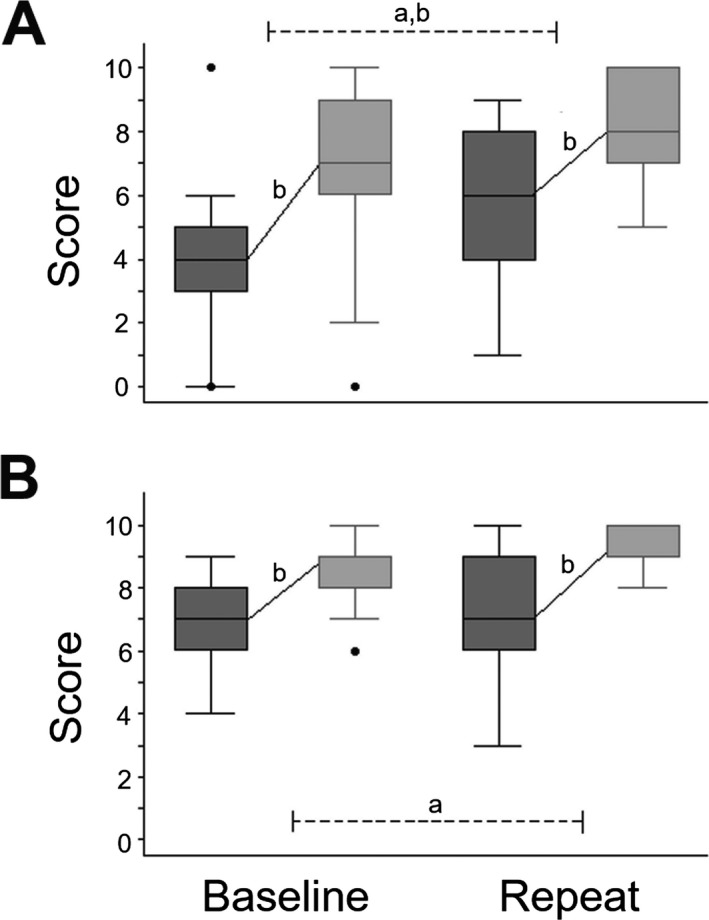
Profiles of sinewave speech comprehension by the participant groups are shown for baseline and repeat behavioral test sessions (see also Table 1). **A:** Alzheimer's disease; **B:** Healthy controls. For each group and test session, dark gray oblongs code interquartile range and whiskers the overall range of scores for the first 10 trials; light gray oblongs code interquartile range and whiskers the overall range of scores for the second 10 trials; solid lines indicate the mean change in scores between trial blocks in each test session; and dotted lines indicate comparisons between sessions. Values falling outside these ranges are indicated. ^a^Significant between‐group difference (*P* < 0.05); ^b^significant within‐group change (*P* < 0.05).

The Table shows mean (standard deviation) general demographic, clinical, and behavioral test data in the healthy control group and Alzheimer's disease (AD) patient group; comparisons between groups are also indicated. All AD patients were established on a standard daily 10 mg dose of donepezil at the time of participation; the baseline session was conducted prior to their next, intersession dose of donepezil. The interval between test sessions was similar for each group (healthy controls, 4.8 ± 0.4 h; AD, 4.9 ± 0.4 h; *t*_32_ = −0.85, *P* = 0.400). The right‐hand columns show the effect of statistical comparisons between participant groups for each test. Maximum scores for standard tests of neuropsychological functions and for tests in the speech experiment (spoken numbers presented in sinewave form and in clear) are indicated in parentheses (see text and Fig. 1 for details). AD, Alzheimer's disease; BPVS, British Picture Vocabulary Scale (Dunn & Whetton, 1982); F, female; GNT, Graded Naming Test (McKenna & Warrington, 1980); L, left; M, male; MMSE, Mini‐Mental State Examination score; NA, not applicable; R, right; RMT, Recognition Memory Test (Warrington, 1984); WASI, Wechsler Abbreviated Scale of Intelligence (Wechsler, 1999).

a no participant was taking memantine or other psychoactive agents;

b one AD patient did not complete the test due to lack of time;

c significant between‐session performance difference within group (*P* < 0.001);

d index of intrinsic perceptual learning of sinewave speech (score on second 10 trials minus score on first 10 trials, within that session; see text and Figure 2).

### Experimental procedures

**S**timuli for the degraded speech experiment comprised spoken three‐digit numbers (of the form, ‘eight hundred and ninety‐seven’), converted after recording to sinewave replicas using standard methods and delivered via headphones (see Fig. 1).

Each participant was assessed in the morning (baseline session) and again the same afternoon (repeat session; mean intersession interval 4.8 h; see Table 1). AD patients began the baseline testing session at least 12 h following their last dose of donepezil and then took their daily 10 mg dose of donepezil between sessions (mean 2.6 ± 0.7 h prior to the repeat session). At each session, all participants completed the test assessing sinewave speech perception, a control test assessing perception of clear (natural) speech and a standard general assessment of cognitive functions (Table 1). The sinewave speech test comprised 20 trials; two different three‐digit number lists were used for the baseline and repeat sessions and the order of these lists was counterbalanced across participants. The clear speech test comprised 10 trials based on a separate three‐digit number list. In both tests, the task on each trial was to transcribe the three‐digit number presented as fully as possible and each trial was scored as correct or incorrect. For each participant at each test session, we calculated a within‐session change score (total score on the second 10 trials minus total score on the first 10 trials): this provided an index of intrinsic perceptual learning (i.e., improved perceptual responsiveness resulting from sensory experience^14^), independent of intervening acetylcholinesterase inhibition.

### Data analyses

All data were analyzed using Stata14.0^®^. Participant groups were compared on demographic and baseline neuropsychological data using two‐tailed t‐tests for continuous variables and chi‐square tests for categorical variables. Neuropsychological performance was compared between baseline and repeat sessions by calculating an intersession change (difference) score for each test in each participant. Change scores (both within‐session and between sessions) were entered as dependent variables in a series of two‐tailed independent sample t‐tests for assessing between‐group differences and two‐tailed one‐sample t‐tests for within‐group differences. Correlations between perceptual performance and general clinical indices in the AD group were assessed using Pearson's correlation. General psychometric data for participant groups were assessed at statistical significance threshold *P* < 0.01 (Bonferroni‐adjusted for multiple comparisons); key sinewave speech comparisons motivated by our specific prior hypothesis were assessed at *P* < 0.05. Where normality assumptions were violated we substituted nonparametric analogues of standard statistics (Wilcoxon rank‐sum for independent sample t‐tests, Wilcoxon signed‐rank for one‐sample t‐tests).

## Results

Participant group profiles and comparisons between groups are presented in Table 1 and Figure 2. Participant groups were well‐matched for age, gender, and other demographic characteristics, and patient groups showed the anticipated profiles of impairment on baseline general neuropsychological assessment. For the healthy control group and AD group considered separately, performance on a range of general neuropsychological functions did not alter significantly between baseline and repeat testing sessions (*z* range = 0.042–1.92, all *P* > 0.01); there was no significant effect of diagnosis on change in performance between sessions.

For both the healthy control group and the AD group, performance on perception of clear speech was essentially at ceiling across test sessions for all participants. Both groups showed perceptual learning of sinewave speech across the baseline test session (AD: *t*_16_ = 3.92, *P* < 0.001; controls: *t*_16_ = 5.66, *P* < 0.001); this intrinsic learning effect did not differ significantly between groups (*t*_16_ = −0.91, *P* = 0.369). However, the AD group was significantly less accurate than the healthy control group on perception of sinewave speech at baseline (*z* = −3.40, *P* < 0.001) and showed a significant improvement between sessions (i.e., following administration of donepezil; *t*_16_ = 4.01, *P* < 0.001) that was not evident in the healthy control group (*t*_16_ = 1.19, *P* = 0.126). This differential improvement was reflected in a significant effect of diagnosis on change in performance (*t*_16_ = −2.36, *P* = 0.025 and evident in 13/17 (76%) of patients in the AD group. Across the repeat test session, both groups again showed comparable perceptual learning of sinewave speech (AD: *t*_16_ = 7.35, *P* < 0.001; controls *t*_16_ = 4.75, *P* < 0.001; group comparison *t*_16_ = −0.19, *P* = 0.849).

The effect of improved sinewave speech perception in the AD group did not correlate with disease severity (Mini‐Mental State Examination score; *r* = 0.02, *P* = 0.931), symptom duration (*r* = 0.23, *P* = 0.378) or duration of donepezil treatment (*r* = 0.04, *P* = 0.889). Across the participant cohort, there were no significant performance differences between the two word lists used in the sinewave speech test (Wilcoxon signed‐rank: *z* = 1.61, *P* = 0.108).

## Discussion

We have shown that (relative to healthy older individuals) patients with AD have impaired perception of degraded (sinewave) speech and that this deficit is ameliorated by acetylcholinesterase inhibition, under clinically relevant dosing conditions. This procholinergic benefit in the AD group did not extend to other cognitive measures over a similar time‐frame and did not correlate with general indices of overall disease severity, arguing for a relatively specific effect on degraded speech perception rather than a generic enhancement of cognitive function or practice effect. Patients with AD showed rates of improvement in understanding sinewave speech comparable to healthy controls both pre‐ and post‐administration of donepezil, suggesting that the intersession benefit was attributable to the drug.

Taken together our findings suggest that, in AD, cholinesterase inhibition acts to amplify the accuracy of sinewave speech perception, whereas intrinsic capacity for perceptual learning of this degraded speech stimulus is retained. These findings are in line with other work demonstrating cholinergic effects on visual perception and cortical function in AD,^8^ with retained endogenous perceptual learning mechanisms.^15^ While the mechanism of the procholinergic effect on degraded speech perception remains undefined, there are several plausible candidates, acting alone or in concert: these include enhanced precision of synaptic transmission and predictive filtering in ascending auditory pathways and facilitation of spectral integration, feature encoding and tracking in auditory cortex.^6^, ^16^, ^17^, ^18^, ^19^, ^20^

Sinewave speech provides a quantifiable metric for speech perception under challenging listening conditions; our findings are potentially relevant to a variety of daily life situations (for example, accented speech, noisy telephone lines, and cocktail party scenarios) that stress speech perception mechanisms and present particular difficulties for patients with AD.^1^, ^2^, ^3^, ^4^ The relative selectivity of the present effect suggests that the pairing of procholinergic therapy with specific, dynamic auditory stimuli such as degraded speech may be required to train and to measure perceptual benefit. Our findings have clear implications for assessing dynamic perceptual reserve, improving communication and designing interventions and acoustic environments from the early stages of AD.^5^ Besides corroboration in larger cohorts using a placebo‐controlled design and drug‐naive patients, future work should address the pharmacological, neurophysiological and neuroanatomical correlates of these findings in relation to peripheral hearing, the circadian alertness cycle and other disease factors and the durability and translatability of sinewave speech effects to other adverse listening paradigms.

## Author Contributions

CJDH, JDW, SJC, BHR, and MNR were responsible for the conception and design of the study. CJDH, YTH, RLB, CRM, and BHR were responsible for the acquisition and analysis of data. CJDH and JDW drafted the manuscript.

## Conflicts of Interest

The authors have no relevant conflicts of interest to declare.
